# Supplementary material for: Phenotypic and transcriptomic responses of cultivated sunflower seedlings (Helianthus annuus L.) to four abiotic stresses
Source: PLoS One. 2022 Sep 30;17(9):e0275462. doi: 10.1371/journal.pone.0275462 (PMC9524668; doi:10.1371/journal.pone.0275462)

Figure S1A: MDS All Samples All Genes

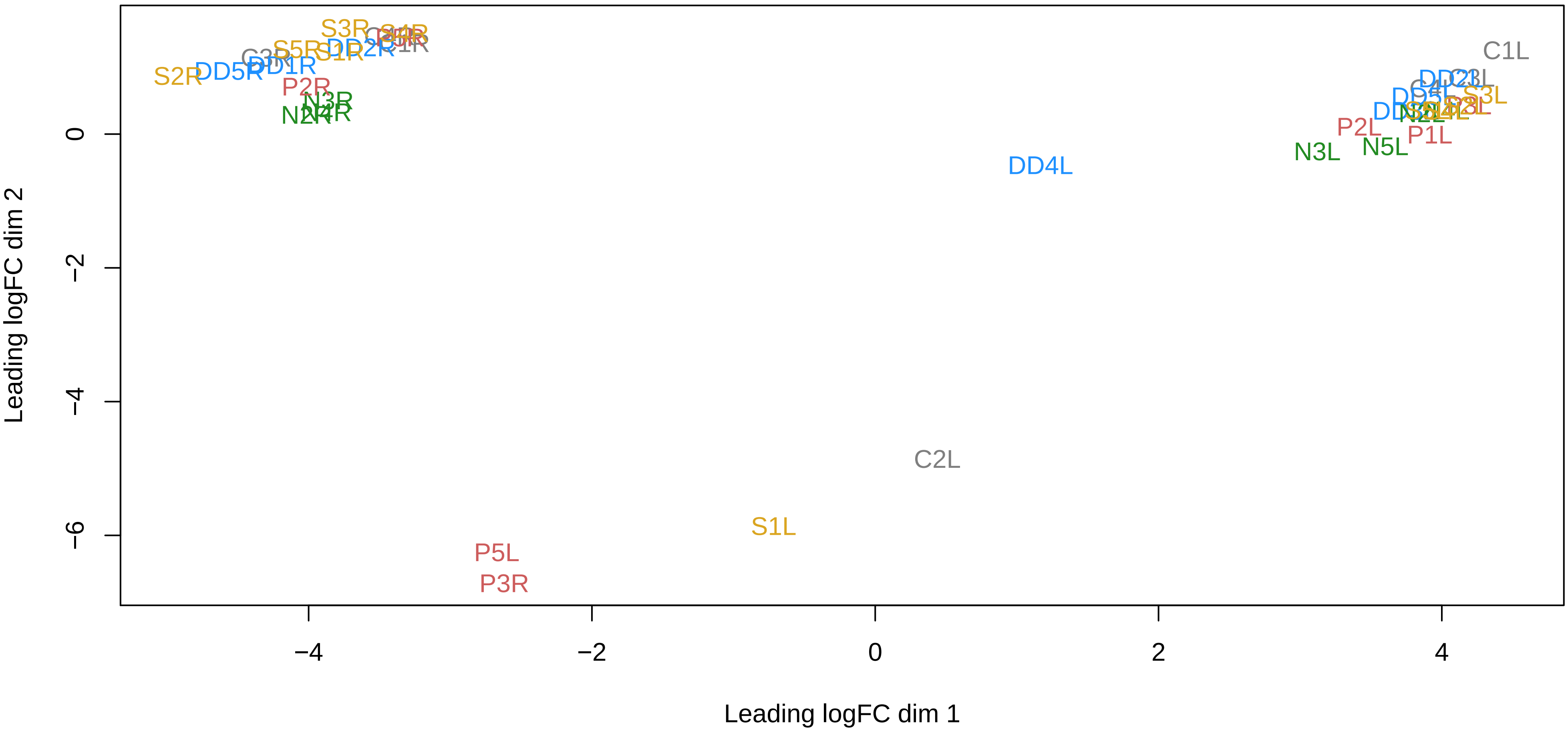

Figure S1B: MDS No Outliers All Genes

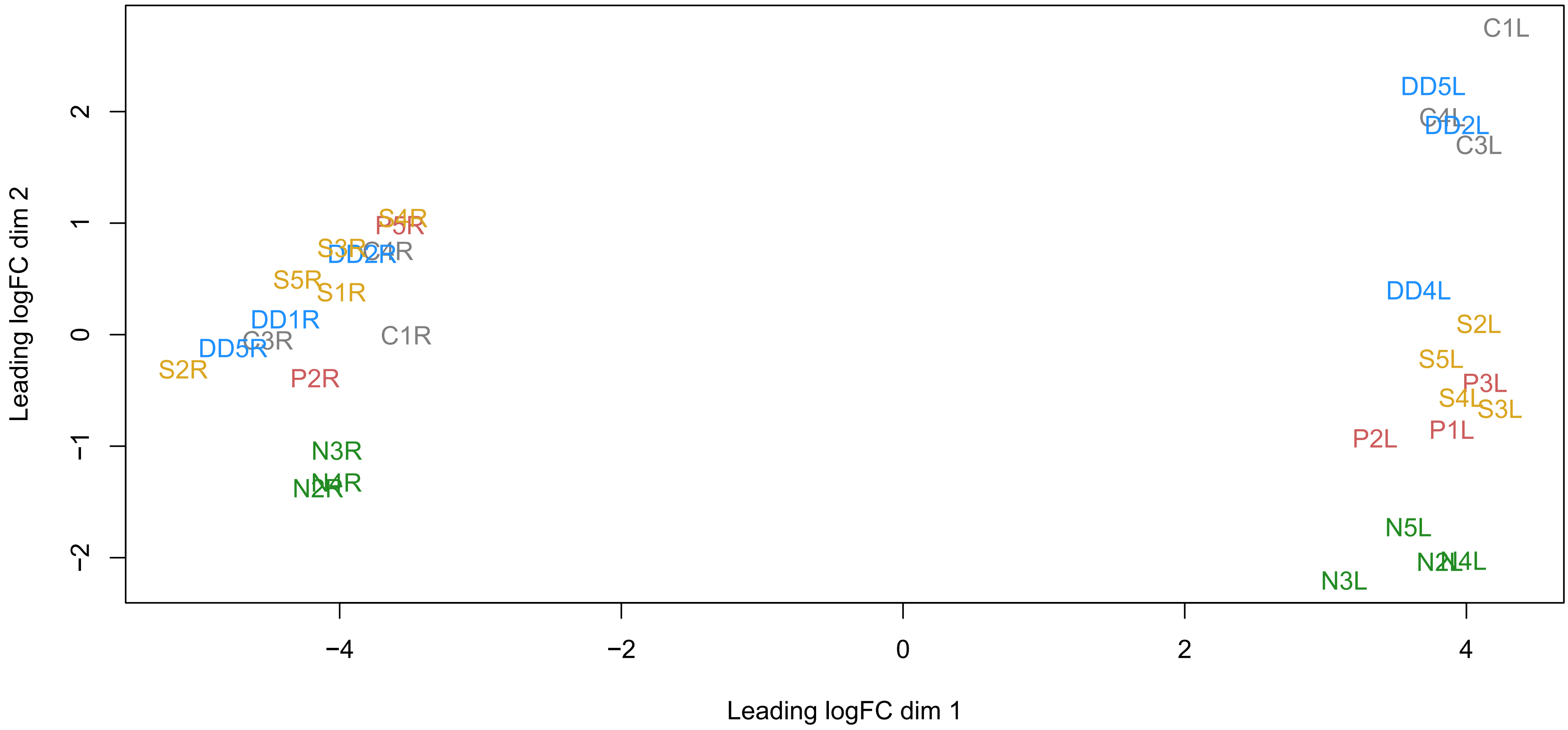

Figure S1C: MDS Leaf Samples All Genes

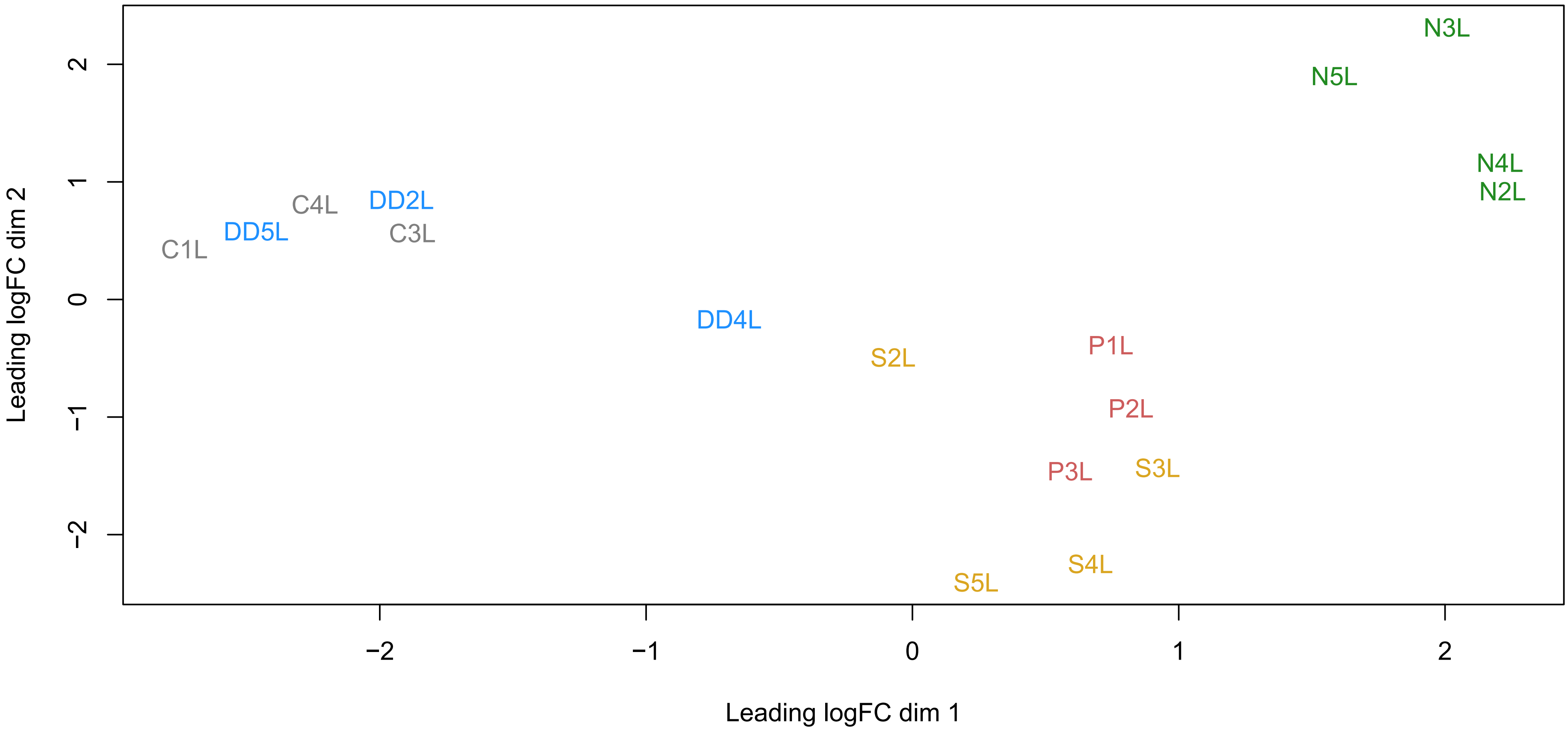

Figure S1D: MDS Leaf Samples No Controls All Genes

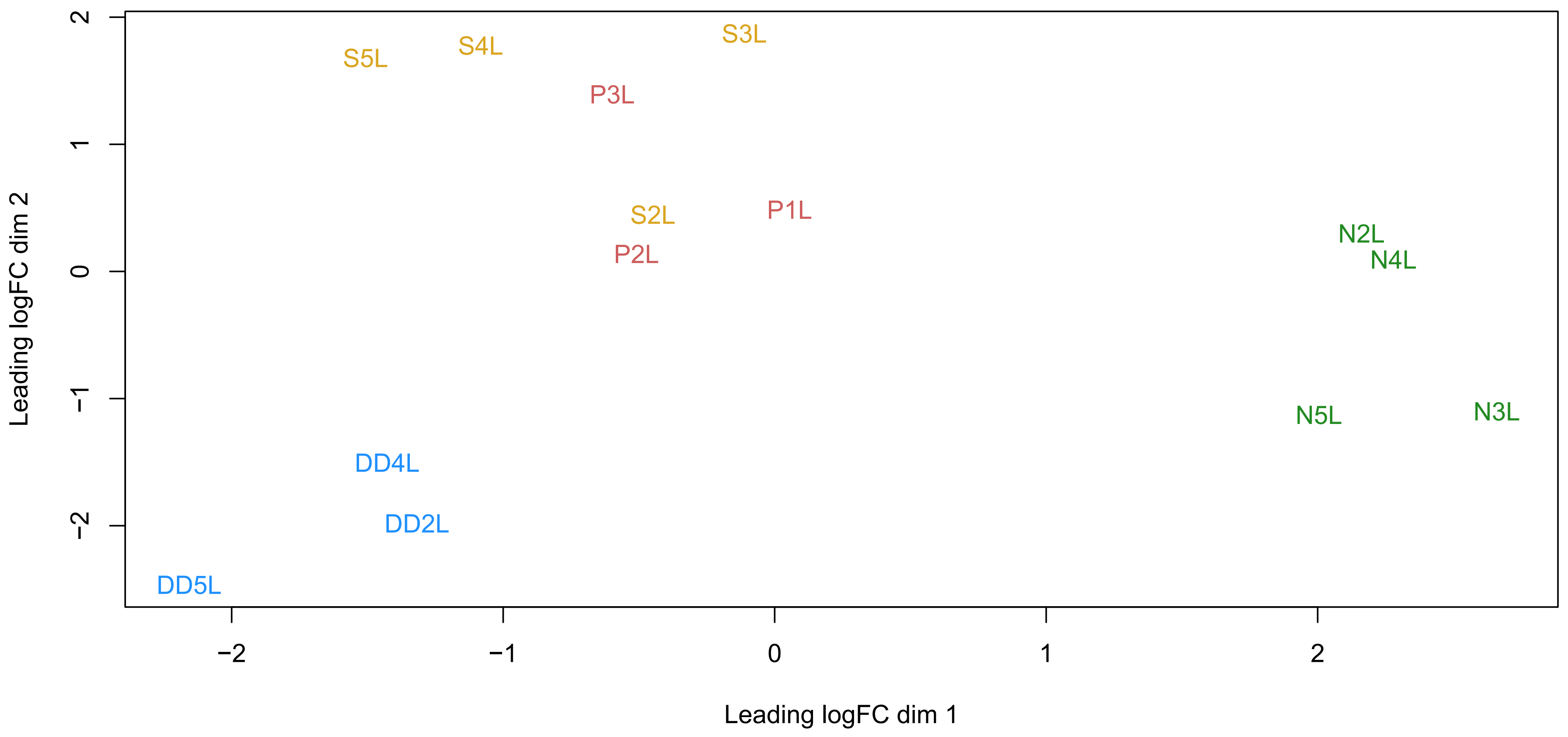

Figure S1E: MDS Root Samples All Genes

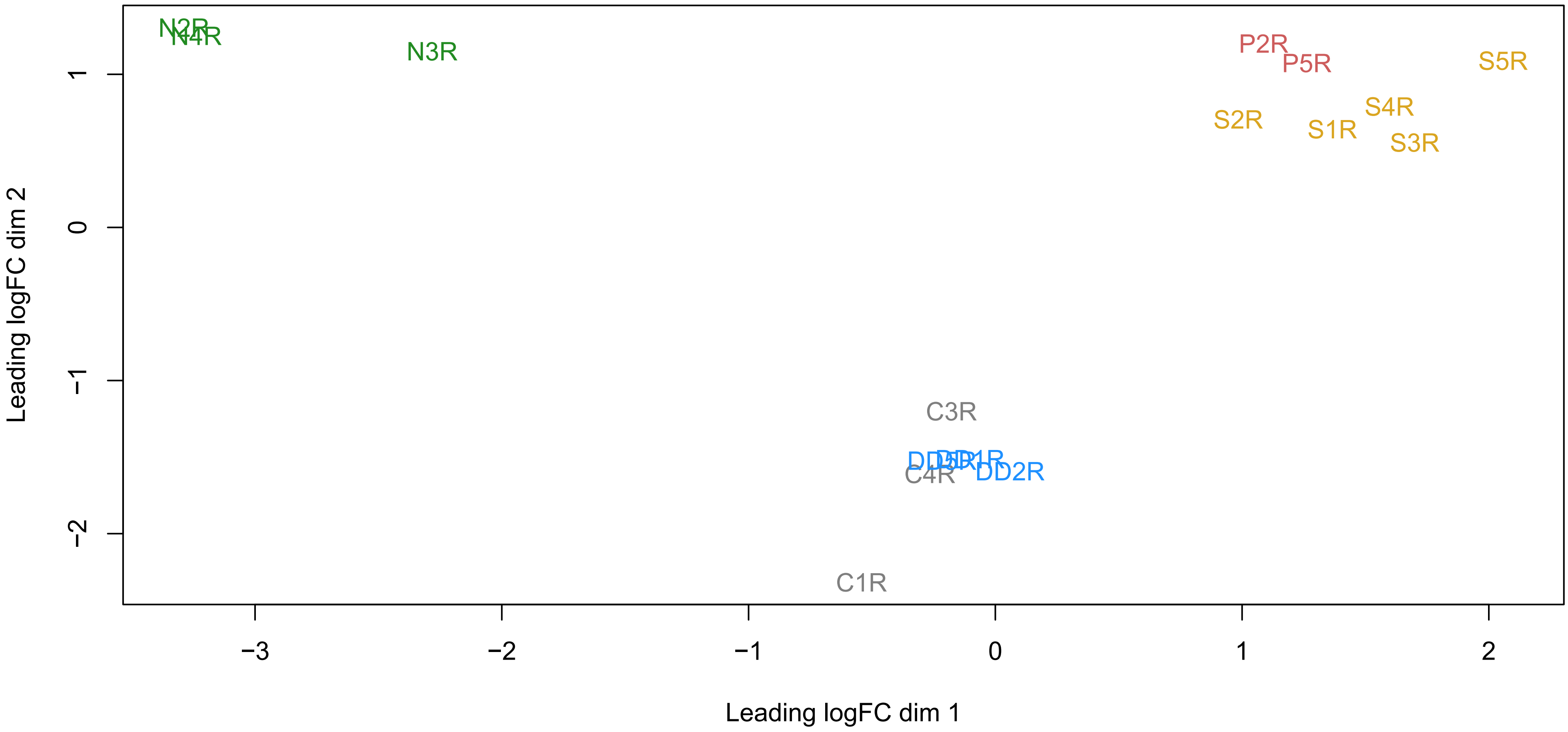

Figure S1F: MDS Root Samples No Controls All Genes

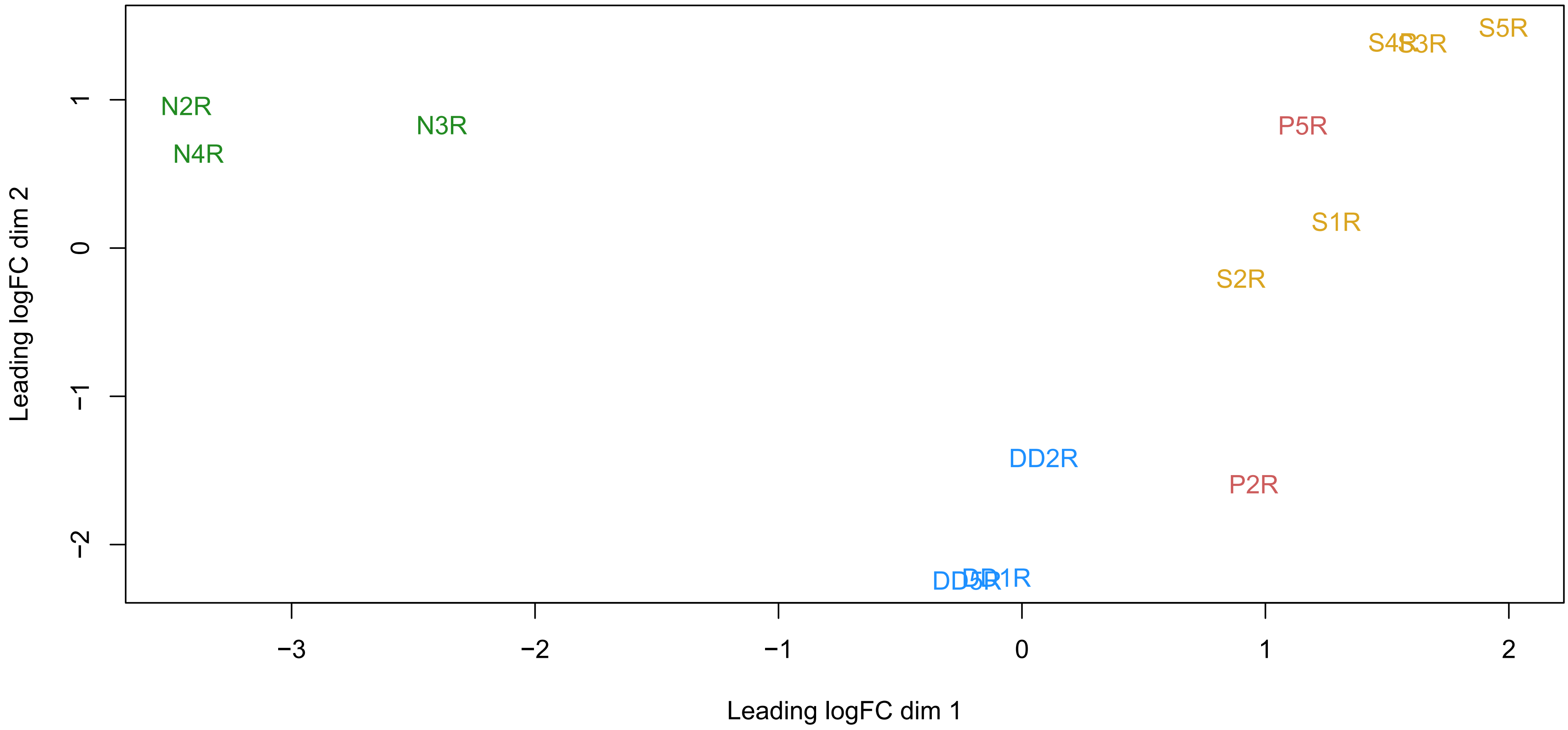

Figure S1G: MDS Leaf Samples All DEGs

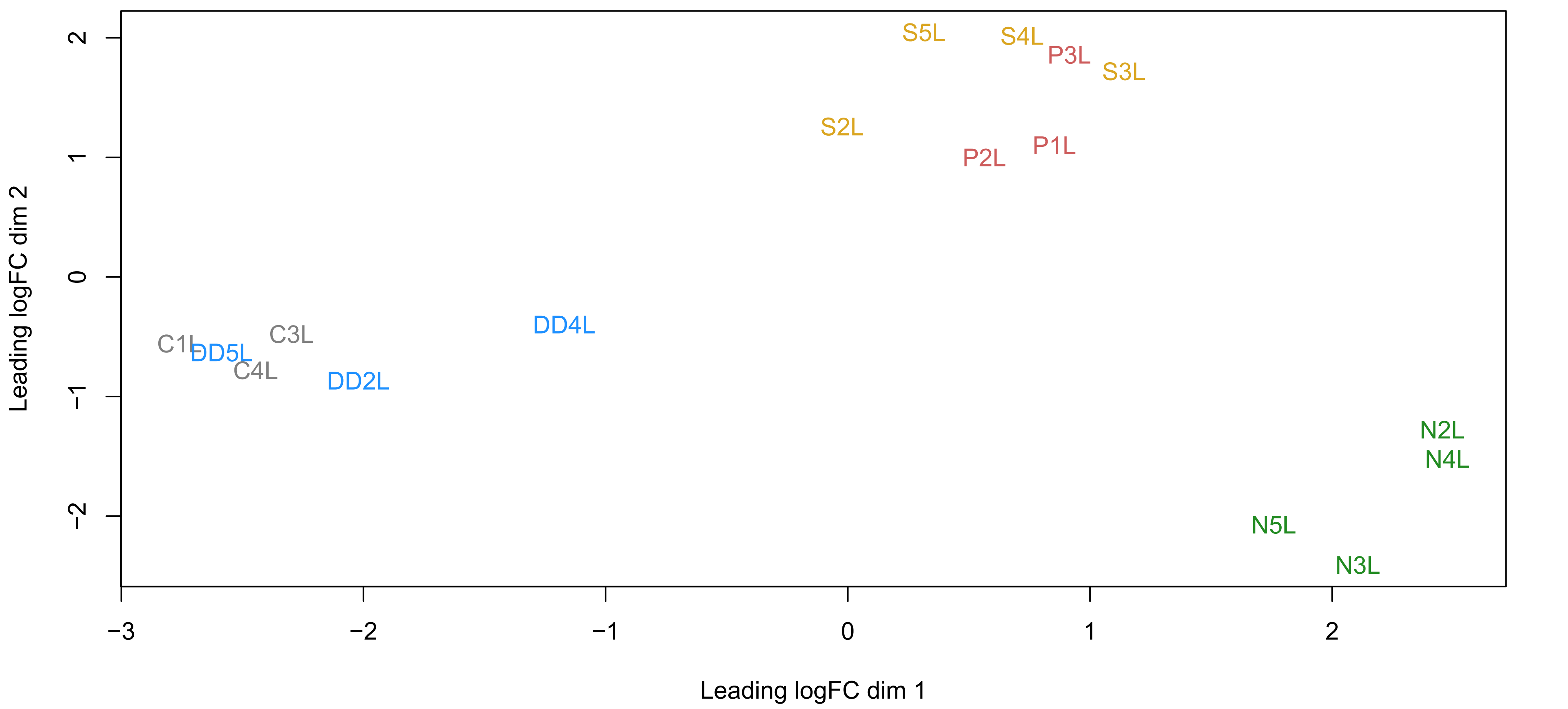

Figure S1H: MDS Leaf Samples No Controls All DEGs

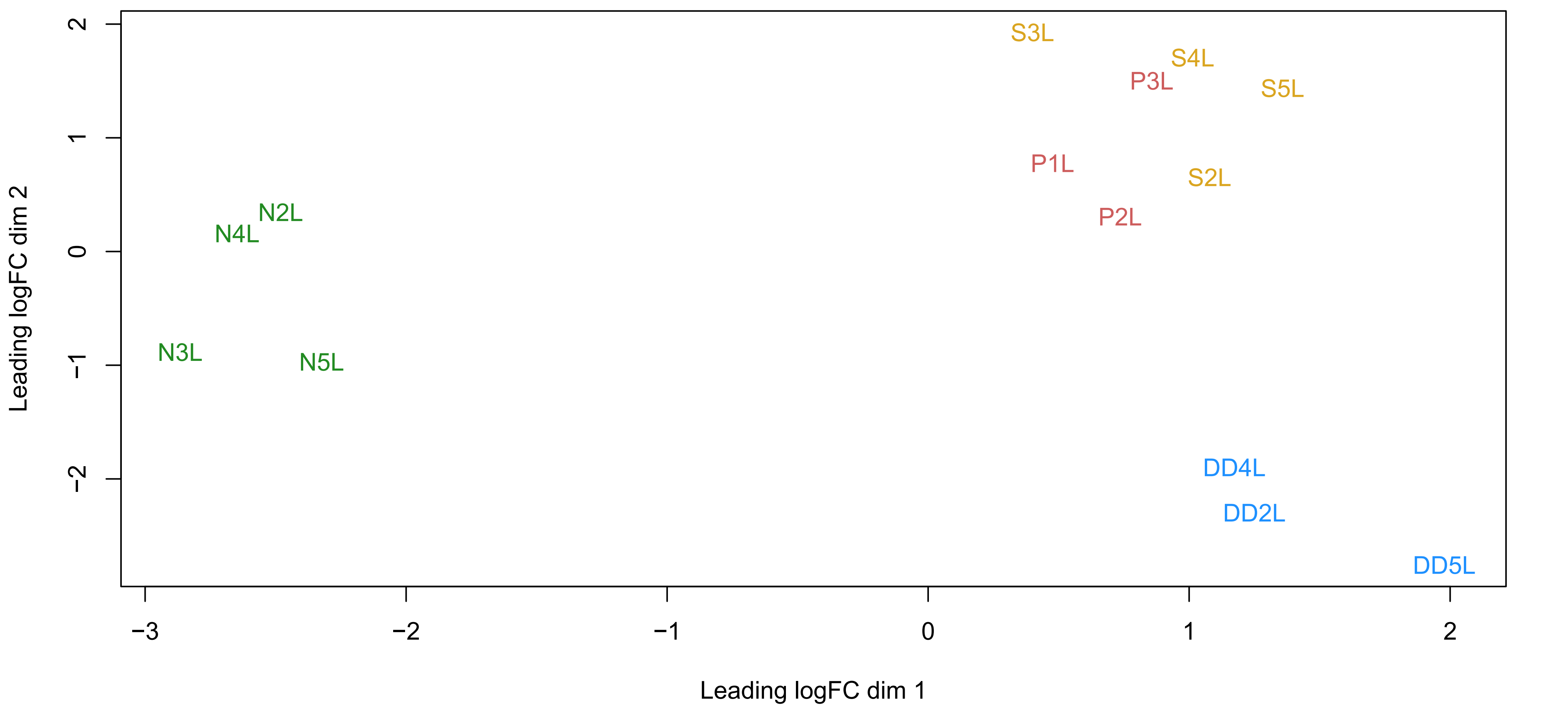

Figure S1I: MDS Root Samples All DEGs

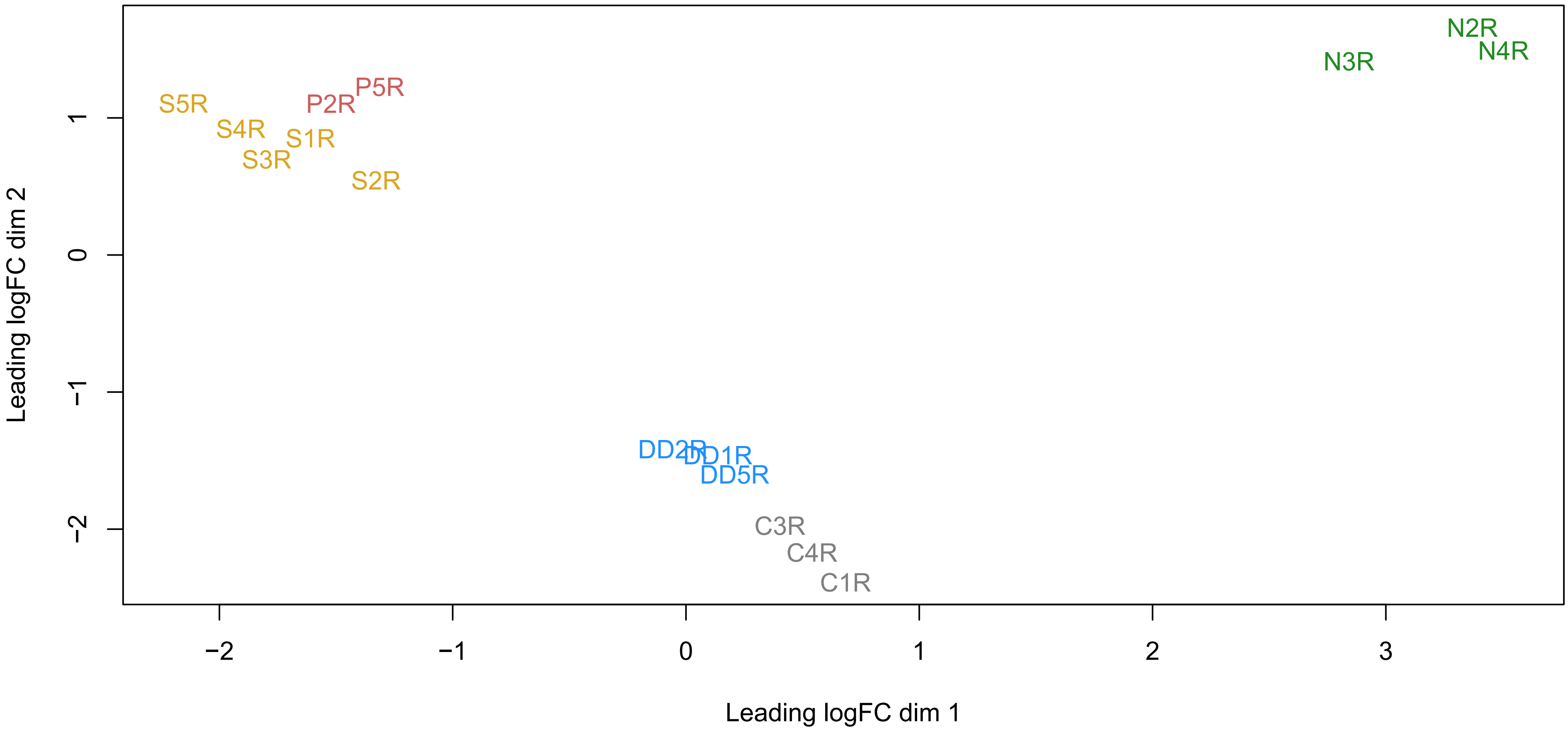

Figure S1J: MDS Root Samples No Controls All DEGs

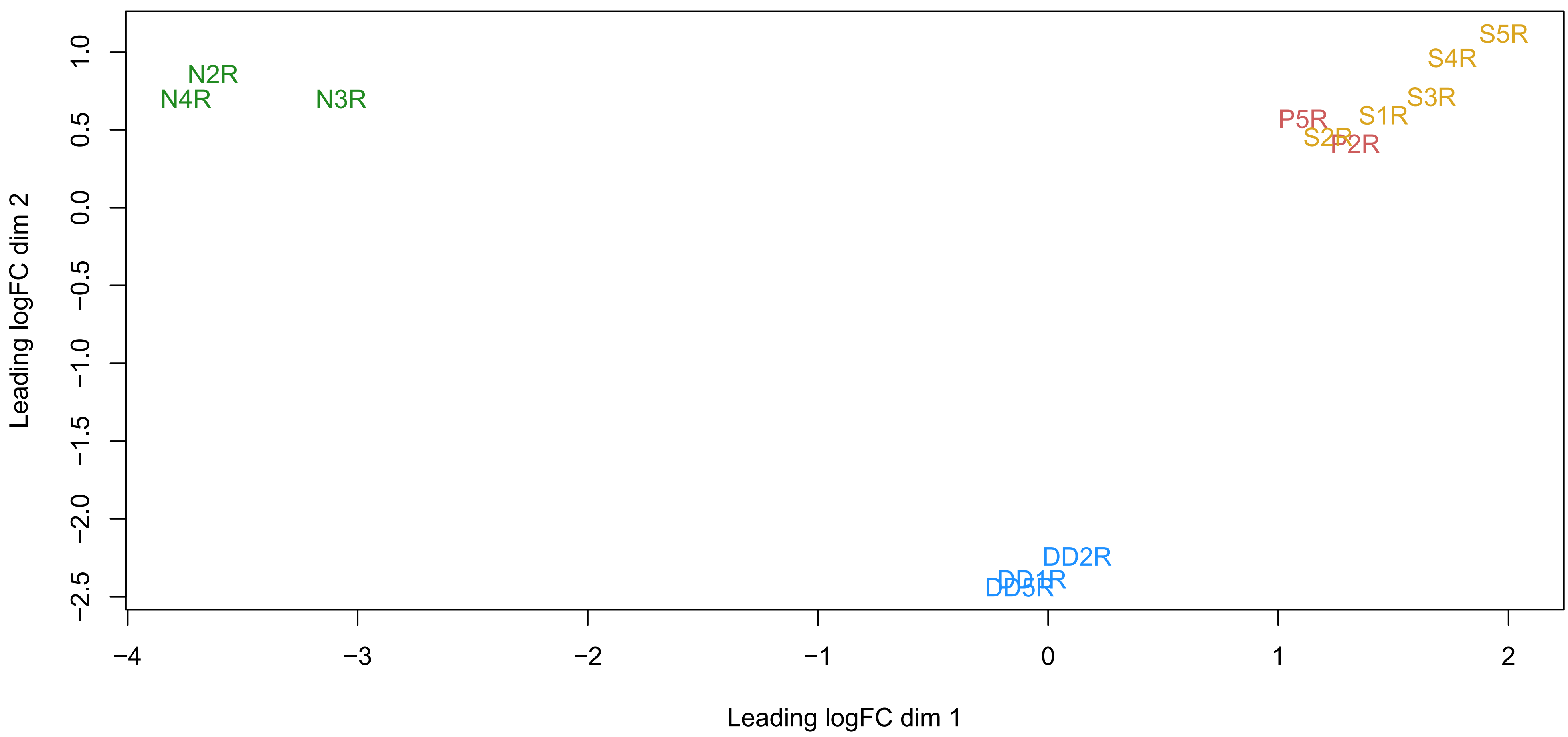

Figure S1K: MDS Leaf Samples Shared DEGs

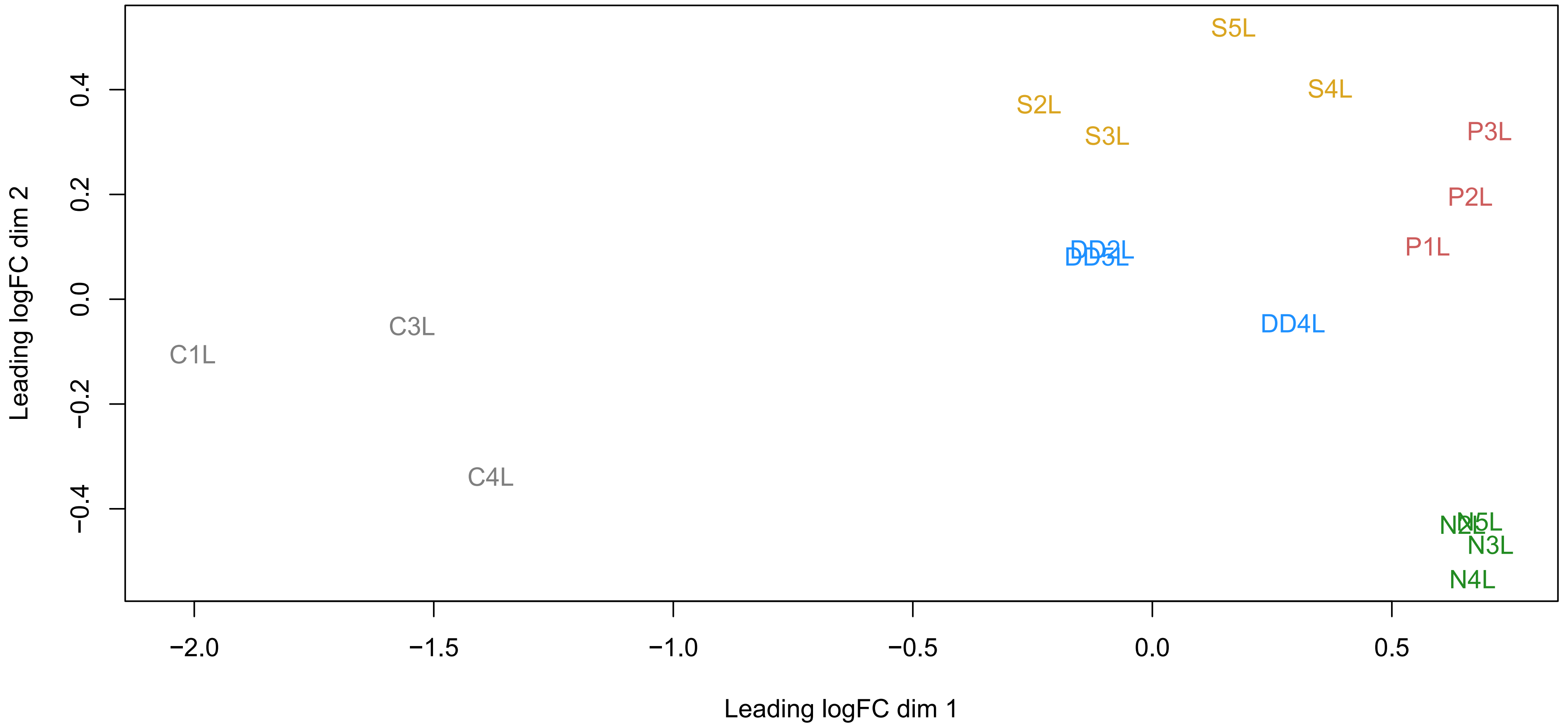

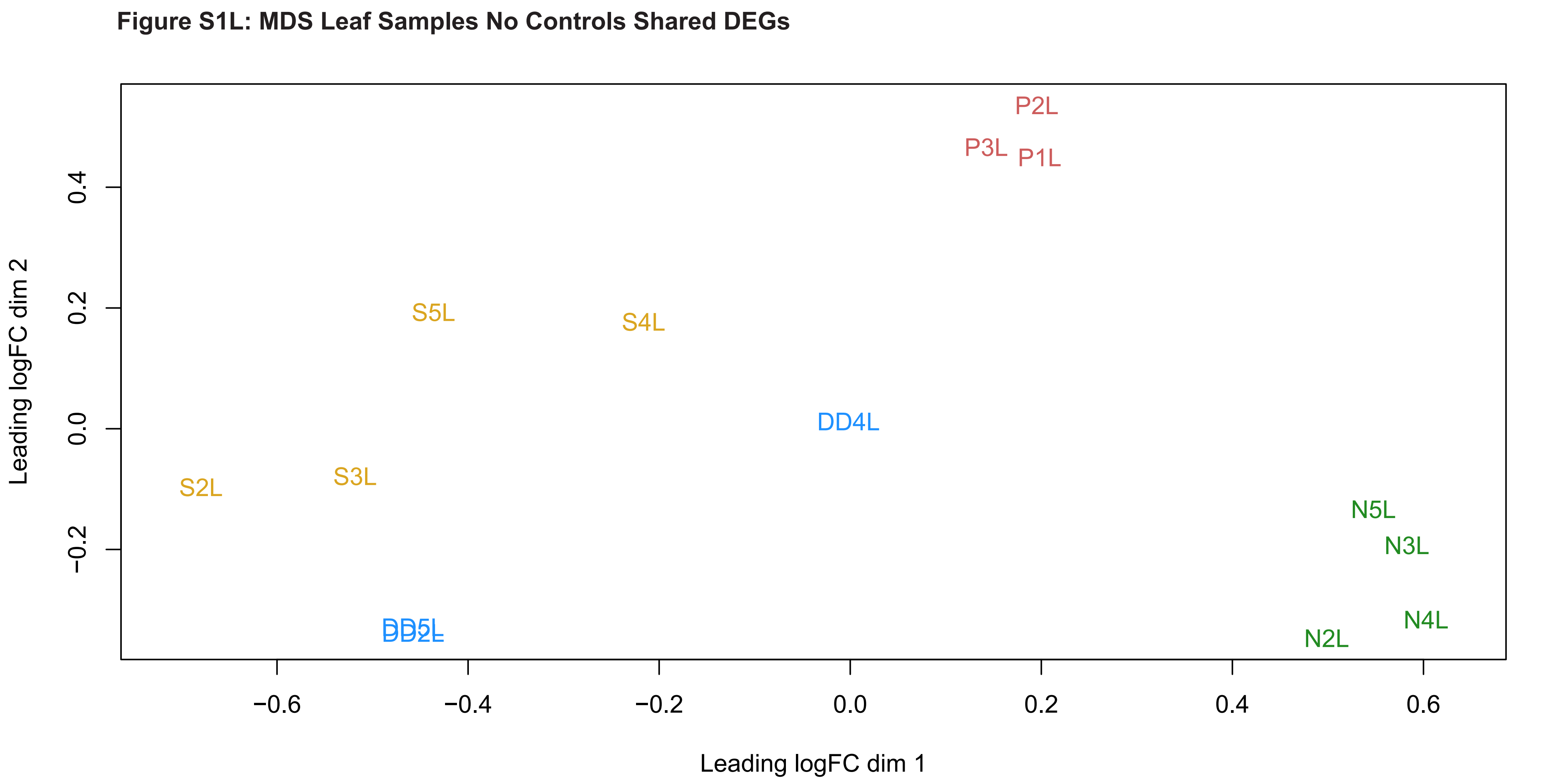

Figure S1L: MDS Leaf Samples No Controls Shared DEGs
P2L
P3L
P1L
S5L
S4L
DD4L
S2L
S3L
N5L
N3L
N4L
N2L
DD5L
DD2L
0.4
0.2
0.0
−0.2
−0.6
−0.4
−0.2
0.0
0.2
0.4
0.6
Leading logFC dim 1
Leading logFC dim 2

Figure S1M: MDS Root Samples Shared DEGs

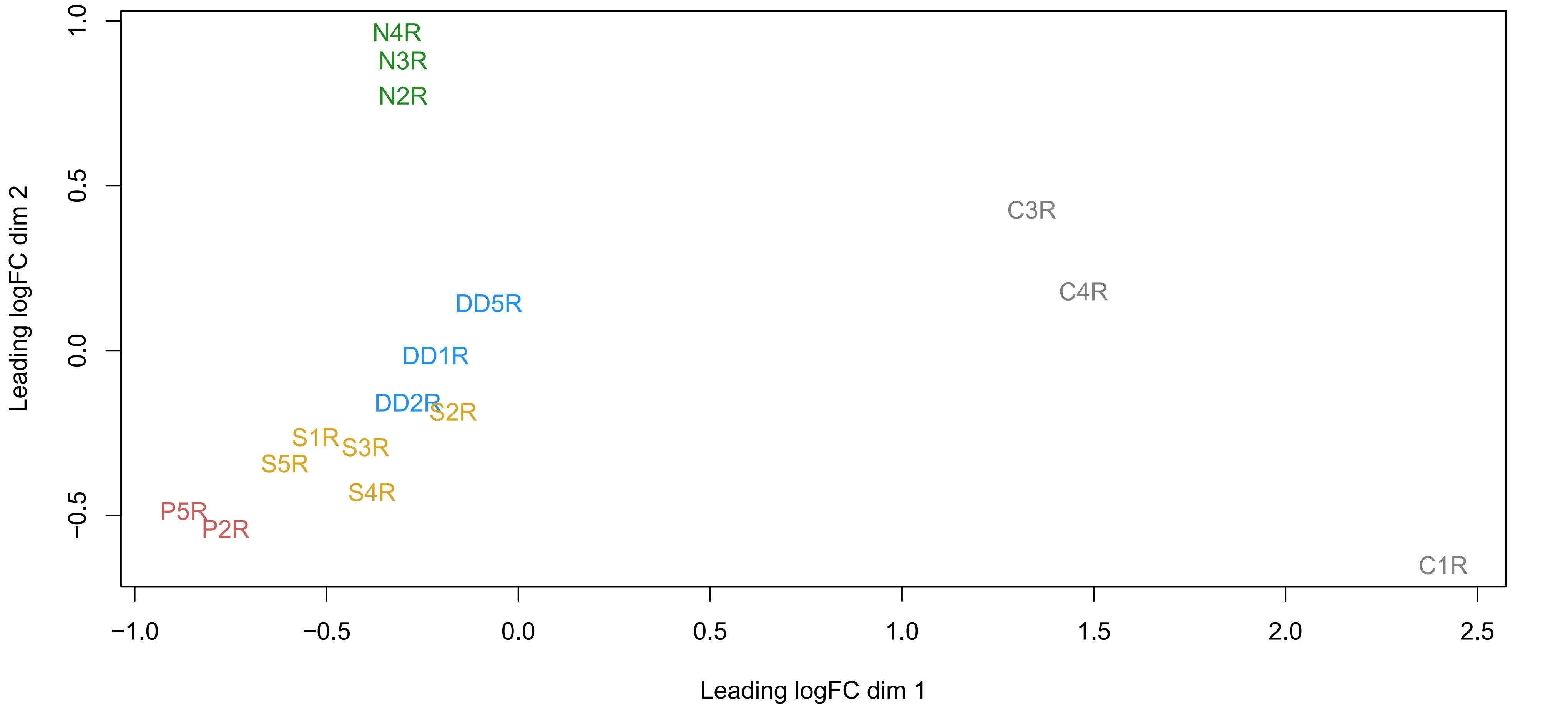

Figure S1N: MDS Root Samples No Controls Shared DEGs

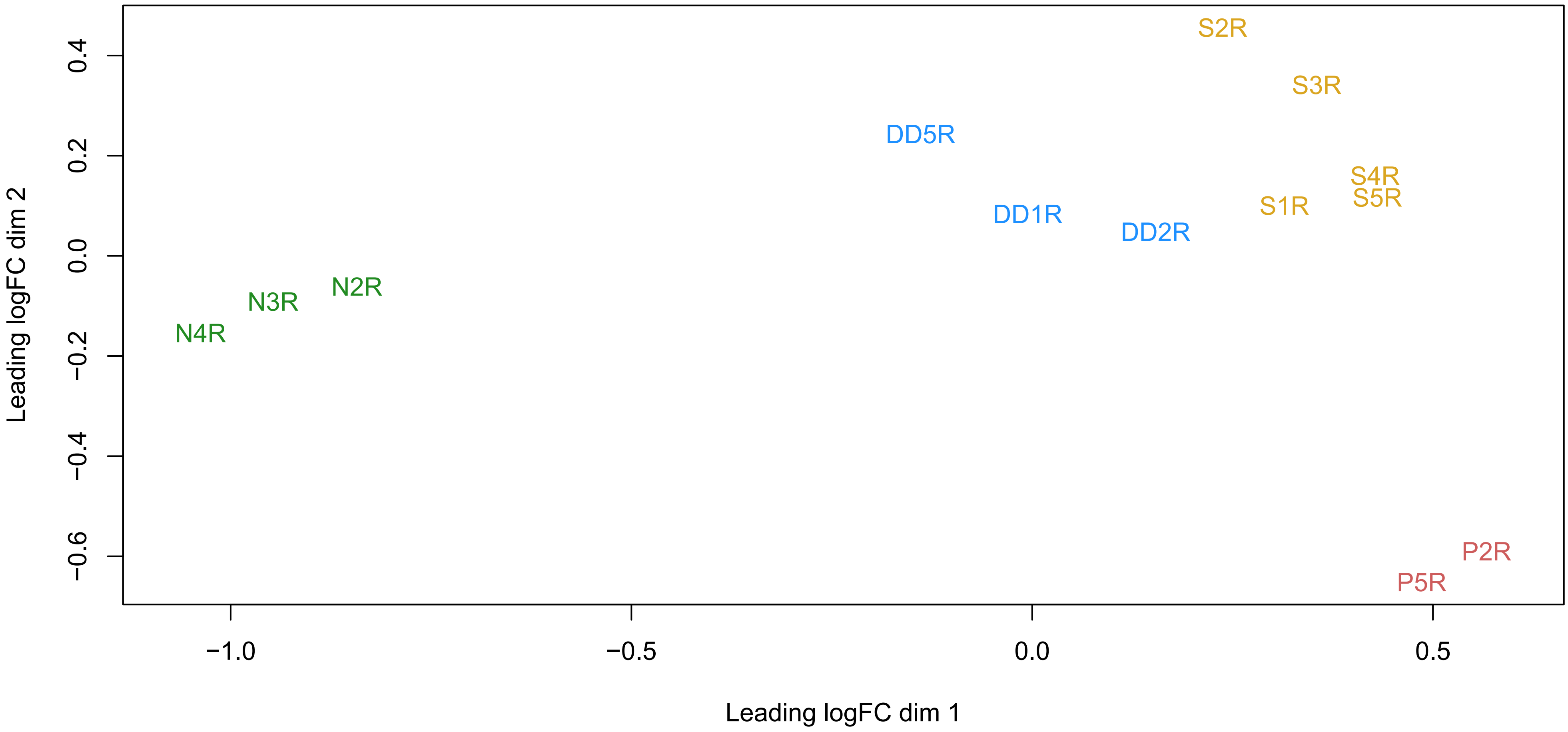

Supplement: S1 Fig — Samples are labeled using a common naming theme: The first letters correspond to the stress treatment (DD = dry-down, P = PEG, S = salt, N = low-nutrient), followed by a number representing the identity of that sample, followed by another letter corresponding to tissue type (L = leaf, R = root). The set of samples and genes used for each MDS plot are described in the label for each subfigure. (PDF) [file pone.0275462.s001.pdf]
