# Supplementary figures and images for: Phenotypic and transcriptomic responses of cultivated sunflower seedlings (Helianthus annuus L.) to four abiotic stresses
Source: PLoS One. 2022 Sep 30;17(9):e0275462. doi: 10.1371/journal.pone.0275462 (PMC9524668; doi:10.1371/journal.pone.0275462)

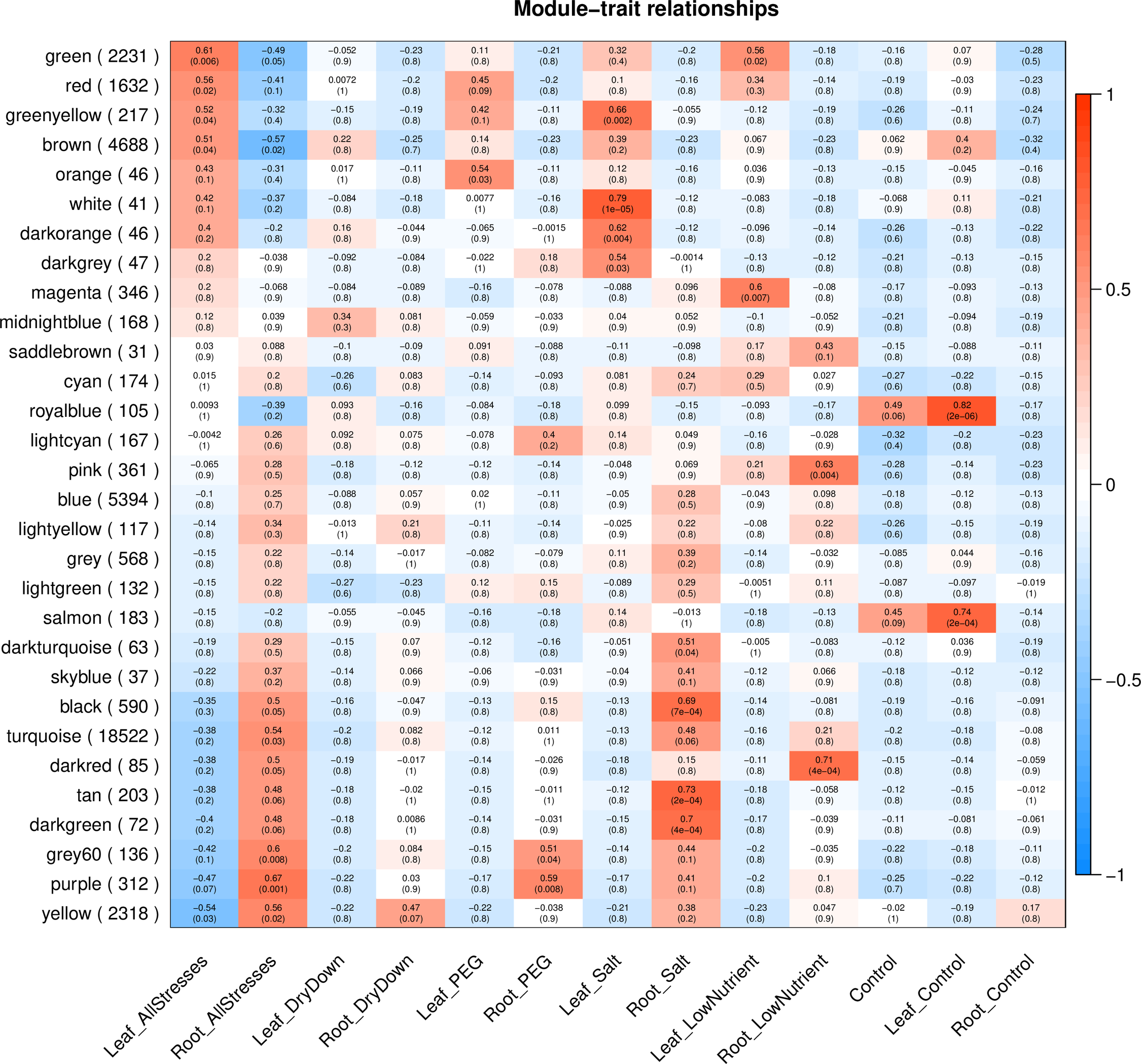

Supplement: S2 Fig — Correlation values (upper text) and P-values (lower, parenthetical text) are presented in each cell. Color is determined by the sign and magnitude of the correlation. Positive correlations (red) indicate genes within a module are upregulated within a stress/tissue combination while negative correlations (blue) indicate that genes within the module are downregulated. (TIF) [file pone.0275462.s002.tif]

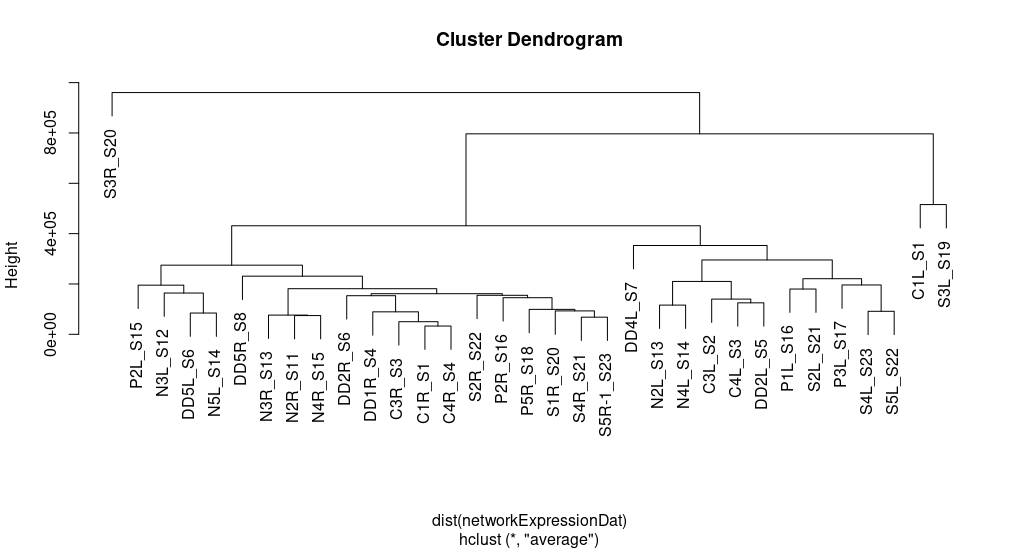

Supplement: S3 Fig — (TIF) [file pone.0275462.s003.tif]
