# Supplementary material for: Phenotypic and transcriptomic responses of cultivated sunflower seedlings (Helianthus annuus L.) to four abiotic stresses
Source: PLoS One. 2022 Sep 30;17(9):e0275462. doi: 10.1371/journal.pone.0275462 (PMC9524668; doi:10.1371/journal.pone.0275462)
Supplement: Attachment — Submitted filename: plos one 20 06 2022.doc [file pone.0275462.s011.doc]

The authors have generated data on phenotypic and transcriptomics using sunflower seedlings under different stresses. The seedlings were grown under dry down, PEG and salinity. These stresses were compared with low nutrient stress for identifying commonality genes through transcriptome studies. The authors worked and generated tremendous data. However, there is no novelty in the work. Hence, It is not recommended for the publication.

Abstract: Too lengthy which was not summarized as per the journal concerns.

61. Climate change is expecting frequent and longer droughts….How this current work tackle the drought stress in plants.

94. Arrange citations in year wise

106. Since many years scientists are claiming the different genes are involving in signaling pathways. Does really help these pathways to protect the plants particularly abiotic stresses?

There is no proper conclusion in the manuscript.
